# Structures of dipotassium rubidium citrate monohydrate, K_2_RbC_6_H_5_O_7_(H_2_O), and potassium dirubidium citrate monohydrate, KRb_2_C_6_H_5_O_7_(H_2_O), from laboratory X-ray powder diffraction data and DFT calculations

**DOI:** 10.1107/S2056989020011846

**Published:** 2020-09-04

**Authors:** Andrew J. Cigler, James A. Kaduk

**Affiliations:** a Department of Chemistry, North Central College, 131 S. Loomis, St., Naperville IL, 60540, USA

**Keywords:** powder diffraction, citrate, potassium, rubidium, density functional theory, crystal structure

## Abstract

The crystal structures of the isostructural compounds dipotassium rubidium citrate monohydrate, K_2_RbC_6_H_5_O_7_(H_2_O), and potassium dirubidium citrate monohydrate, KRb_2_C_6_H_5_O_7_(H_2_O), have been solved and refined using laboratory X-ray powder diffraction data, and optimized using density functional techniques. The compounds are isostructural to K_3_C_6_H_5_O_7_(H_2_O) and Rb_3_C_6_H_5_O_7_(H_2_O), but exhibit different degrees of ordering of the K and Rb cations over the three metal-ion sites. The K and Rb site occupancies correlate well to both the bond-valence sums and the DFT energies of ordered cation systems. The *M*O_6_ and *M*O_7_ coordination polyhedra share edges to form a three-dimensional framework. The water mol­ecule acts as a donor in two strong charge-assisted O—H⋯O hydrogen bonds to carboxyl­ate groups. The hydroxyl group of the citrate anion forms an intra­molecular hydrogen bond to one of the central carboxyl­ate oxygen atoms.

## Abstract 2

The crystal structures of the isostructural mixed-cation compounds dipotassium rubidium citrate monohydrate and potassium dirubidium citrate monohydrate have been solved and refined using laboratory X-ray powder diffraction data and optimized using density functional techniques.

## Chemical context   

A systematic study of the crystal structures of Group 1 (alkali metal) citrate salts has been reported in Rammohan & Kaduk (2018 ▸). The study was extended to lithium metal hydrogen citrates in Cigler & Kaduk (2018 ▸), to sodium metal hydrogen citrates in Cigler & Kaduk (2019*a* ▸), to sodium dirubidium citrates in Cigler & Kaduk (2019*b* ▸), to dilithium potassium citrate (Cigler & Kaduk, 2019*c* ▸), to lithium dipotassium citrate monohydrate in Cigler & Kaduk (2020 ▸), and to potassium rubidium hydrogen citrate in Gonzalez *et al.* (2020 ▸). These compounds represent further extensions to potassium rubidium citrates. The crystal structure of K_3_C_6_H_5_O_7_(H_2_O), Cambridge Structural Database refcode ZZZHVI* has been reported multiple times (Burns & Iball, 1954 ▸; Carrell *et al.*, 1987 ▸), and the structure of Rb_3_C_6_H_5_O_7_(H_2_O) has been reported by Rammohan & Kaduk (2017 ▸).
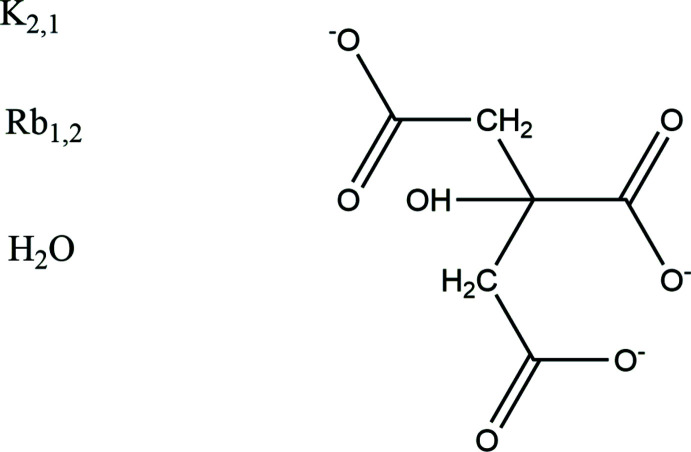


## Structural commentary   

**Figure 1:**
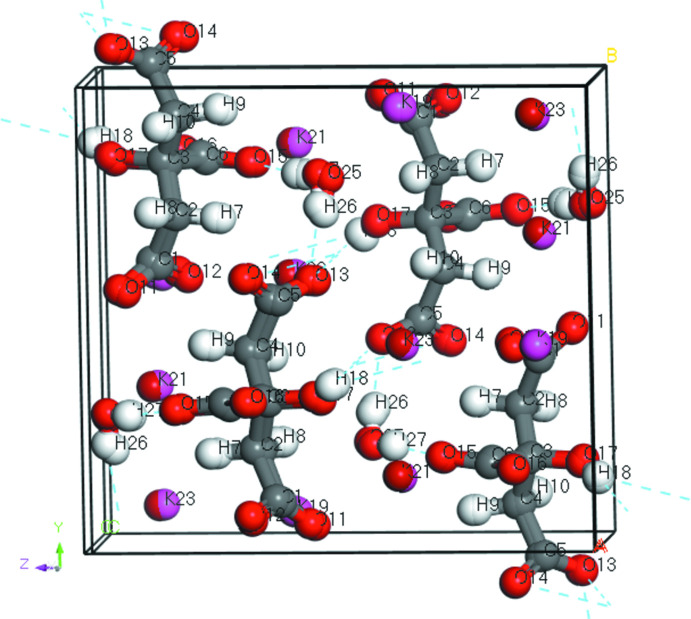
Overlay of the crystal structures of (I) and (II), viewed approximately down the *a*-axis direction.

**Figure 2:**
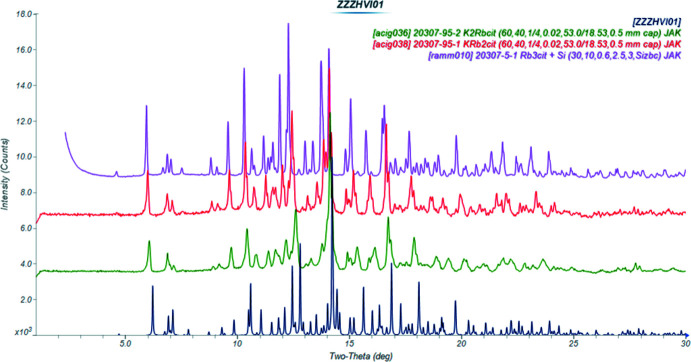
Comparison of the X-ray powder patterns (Mo K_α_ radiation) of K_3_C_6_H_5_O_7_(H_2_O), K_2_RbC_6_H_5_O_7_(H_2_O), KRb_2_C_6_H_5_O_7_(H_2_O), and Rb_3_C_6_H_5_O_7_(H_2_O).

**Figure 3:**
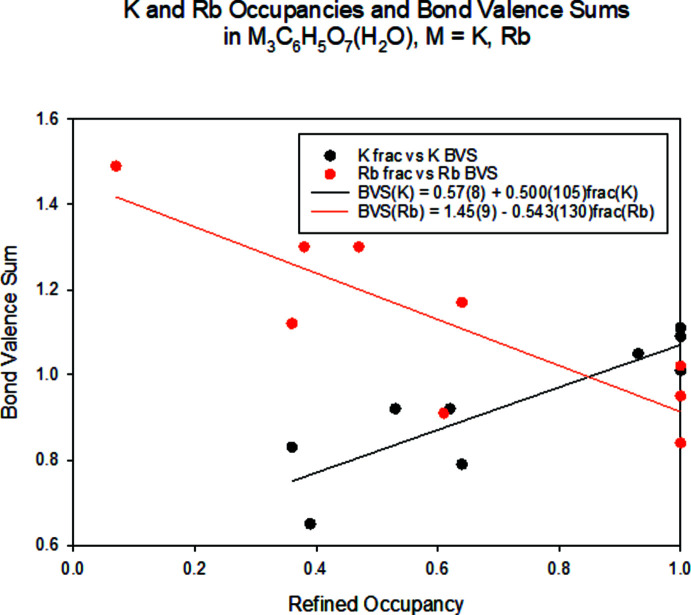
Correlations between the refined K and Rb site occupancies in (K,Rb)_3_C_6_H_5_O_7_(H_2_O) and the bond valence sums for each cation at each of the three potential sites.

The crystal structures of dipotassium rubidium citrate monohydrate K_2_RbC_6_H_5_O_7_(H_2_O), (I), and potassium dirubidium citrate monohydrate KRb_2_C_6_H_5_O_7_(H_2_O), (II), have been solved and refined using laboratory X-ray powder diffraction data, and optimized using density functional techniques. The two compounds are isostructural (Fig. 1 ▸). The powder patterns (Fig. 2 ▸) and the unit cells show that these compounds are isostructural to K_3_C_6_H_5_O_7_(H_2_O) and Rb_3_C_6_H_5_O_7_(H_2_O). In each compound, the K and Rb cations are disordered over the three cation sites: in (I), the K/Rb site occupancies are 0.93/0.07, 0.64/0.36, and 0.53/0.47 for the K19/Rb20, K21/Rb22 and K23/Rb24 sites, respectively and in (II) the refined K/Rb occupancies are 0.62/0.38, 0.39/0.61 and 0.36/0.64 for the same metal sites. The refined site occupancies correlate well to the bond-valence sums calculated for K and Rb at each cation site (Fig. 3 ▸). DFT calculations on ordered cation systems show that in (I) occupation of site 19 by Rb is disfavored by 0.19 kcal mol^−1^, while in (II) occupation of this site by K is favored by 0.28 kcal mol^−1^. These trends are consistent with the refined occupancies, but the energy differences are within the expected errors for such calculations.

**Figure 4:**
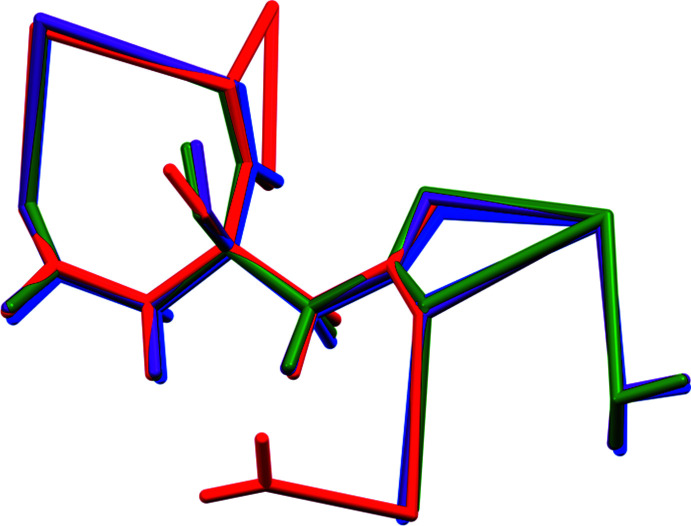
Comparison of the refined asymmetric unit of (I) (red) and the DFT-optimized structures with Rb at site 19 (blue), site 20 (green), and site 21 (purple).

**Figure 5:**
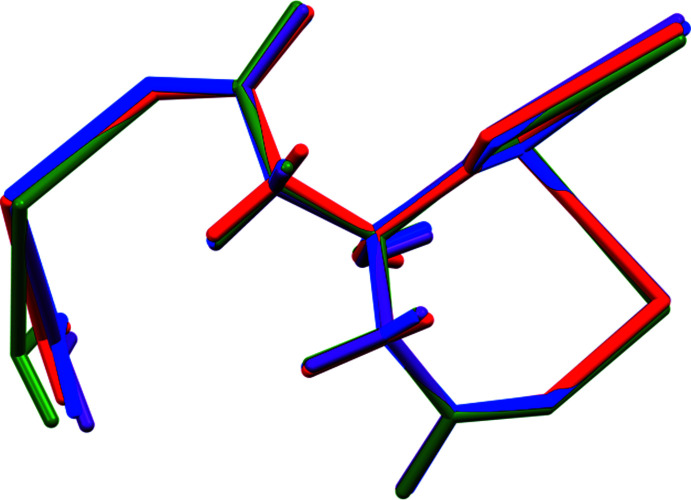
Comparison of the refined asymmetric unit of (II) (red) and the DFT-optimized structures with K at site 19 (blue), site 20 (green), and site 21 (purple).

For (I), the root-mean-square Cartesian displacement of the non-H atoms of the citrate anion in the disordered refined structure and the ordered DFT-optimized structures is 0.114, 0.080, and 0.079 Å for Rb at site 19, 20, and 21 (Fig. 4 ▸). The average absolute difference in the cation positions is 0.085 (29) Å, and the average absolute difference in the position of the water oxygen atom is 0.26 (11) Å. For (II), the similar r.m.s. citrate-atom displacements are 0.077, 0.104, and 0.101 Å (Fig. 5 ▸). The average absolute difference in the cation positions is 0.084 (54) Å, and the average absolute difference in the position of the water mol­ecule oxygen atom is 0.28 (14) Å. The good agreement between the disordered refined structures and the ordered DFT-optimized structures provides confidence that the experimental structures are correct (van de Streek & Neumann, 2014 ▸).

Most of the citrate anion bond distances, bond angles and torsion angles in the experimental structures fall within the normal range indicated by a *Mercury* Mogul Geometry Check (Macrae *et al.*, 2020 ▸). Only the O12—C1—C2 [113.9 (5) and 114.7 (5)°; average = 124 (3)°, *Z*-score = 3.6 and 3.3] and the O13—C5—C4 angles [114.4 (5) and 115.1 (5)°; average = 124 (5)°, *Z*-score = 5.1 and 4.8] are flagged as unusual. The citrate anion occurs in the *trans, trans*-conformation (about C2—C3 and C3—C4), which is one of the two low-energy conformations of an isolated citrate anion (Rammohan & Kaduk, 2018 ▸) and is typical for citrate salts of the larger Group 1 cations. The central carboxyl­ate group and the hydroxyl group exhibit small twist angles [O17—C3—C6—O16 torsion angle = −6 (2) and 0.5 (2)°] from the normal planar arrangement. The Mulliken overlap populations indicate that the K—O and Rb—O bonds are ionic. *M*19/20 is six-coordinate, and *M*21/22 and *M*23/24 are seven-coordinate. The water mol­ecule coordinates to *M*19/20 and *M*21/22.

There is extensive chelation of the citrate anion to the metal ions. The carboxyl­ate groups O11/O12 and O15/O16 chelate to separate metal cations 21/22. The terminal carboxyl­ate O12 and central carboxyl­ate O15 and O16 oxygen atoms chelate to M23/24 and M19/20. The terminal carboxyl­ate O14 and the central carboxyl­ate O15 and O16 chelate to *M*19/20 and *M*23/24. The hydroxyl O17 and terminal carboxyl­ate O11 and O13 chelate to *M*21/22 and *M*23/24.

The Bravais–Friedel–Donnay–Harker (Bravais, 1866 ▸; Friedel, 1907 ▸; Donnay & Harker, 1937 ▸) method suggests that we might expect blocky morphology for these two compounds. No preferred orientation model was necessary in the refinement.

## Supra­molecular features   

**Figure 6:**
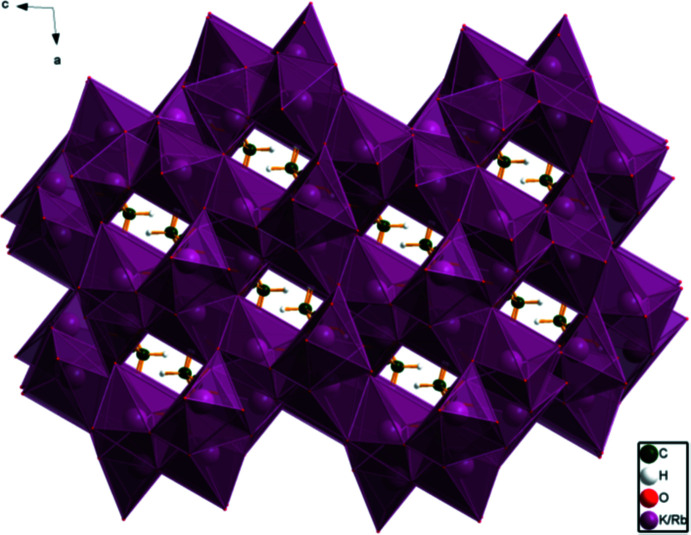
Crystal structure of K_2_RbC_6_H_5_O_7_(H_2_O) and KRb_2_C_6_H_5_O_7_(H_2_O) (shown for K_2_RbC_6_H_5_O_7_(H_2_O)), viewed down the *b*-axis.

The *M*O_6_ and *M*O_7_ coordination polyhedra in both structures share edges to form a three-dimensional framework (Fig. 6 ▸). The hydro­phobic methyl­ene group sides of the citrate anions occupy channels in the framework. The hydrogen bonds in the six ordered systems used for the DFT calculations differ slightly but the general pattern is similar: Tables 1 ▸–3 ▸ ▸ list the geometrical data for (I) with the Rb atom placed at the *M*19, *M*21 and *M*23 sites, respectively and the K atoms occupying the other two sites. Tables 4 ▸–6 ▸ ▸ present data for (II) with the K atom occupying the *M*19, *M*21 and *M2*3 sites, respectively and the Rb atoms occupying the other two sites. The water mol­ecule O25/H26/H27 forms strong charge-assisted hydrogen bonds to the central carboxyl­ate oxygen atom O15 and the terminal carboxyl­ate O13. The energies of the O—H⋯O hydrogen bonds were calculated using the correlation of Rammohan & Kaduk (2018 ▸). The hydroxyl group O17 forms an intra­molecular hydrogen bond to the central carboxyl­ate O16. In some of the ordered models, the hydroxyl group also forms an inter­molecular hydrogen bond to the terminal carboxyl­ate O13.

## Database survey   

Details of the comprehensive literature search for citrate structures are presented in Rammohan & Kaduk (2018 ▸). The powder pattern of K_2_RbC_6_H_5_O_7_(H_2_O) was indexed on a primitive monoclinic unit cell having *a* = 7.2676, *b* = 11.8499, *c* = 13.1006 A, β = 98.234°, *V* = 1116.61 Å^3^ using *DICVOL14* (Louër & Boultif, 2014 ▸). A similar cell was obtained using *N-TREOR* (Altomare *et al.*, 2013 ▸). Analysis of the systematic absences using *EXPO2014* (Altomare *et al.*, 2013 ▸) suggested the space group of *P2*_1_/*n*. The pattern of KRb_2_C_6_H_5_O_7_(H_2_O) was indexed on a similar unit cell using *N-TREOR*, so the compounds were assumed to be isostructural. Reduced cell searches in the Cambridge Structural Database (Groom *et al.*, 2016 ▸) yielded ten10 hits, including four for K_3_C_6_H_5_O_7_(H_2_O), ZZZHVI* (Burns & Iball, 1954 ▸; Carrell *et al.*, 1987 ▸; Rammohan & Kaduk, 2018 ▸).

## Synthesis and crystallization   

Dipotassium rubidium citrate monohydrate, (I), was synthesized by adding stoichiometric qu­anti­ties of 1.382 g K_2_CO_3_ (Sigma–Aldrich) and 1.154 g Rb_2_CO_3_ (Sigma–Aldrich) to a solution of 2.03 g citric acid monohydrate (10.0 mmol, Sigma–Aldrich) in 10 ml of water. After the fizzing subsided, the clear solution was dried in a 403 K oven to yield a white solid. Potassium dirubidium citrate monohydrate, (II), was synthesized in the same way starting from 0.691 g of K_2_CO_3_ and 2.309 g of Rb_2_CO_3_.

## Refinement   

**Figure 7:**
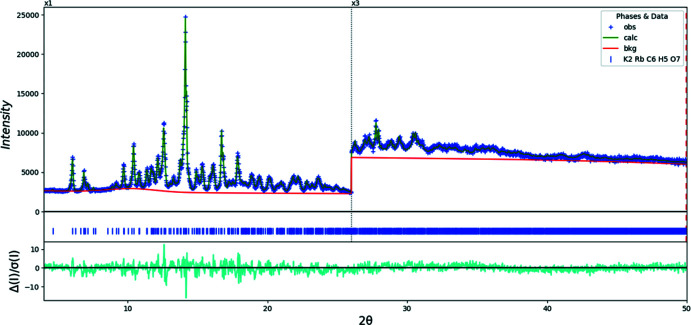
Rietveld plot for (I). The blue crosses represent the observed data points, and the green line is the calculated pattern. The cyan curve is the normalized error plot. The vertical scale has been multiplied by a factor of 3× for 2θ > 26.0°. The row of blue tick marks indicates the calculated reflection positions. The red line is the background curve.

Crystal data, data collection and structure refinement details for (I) are summarized in Table 7 ▸ (Fig. 7 ▸). To minimize Rb fluorescence, the pulse height discriminator lower level of the X’Celerator detector was raised from the default 39.0% to 51.0%. The structure was solved with *FOX* (Favre-Nicolin & Černý, 2002 ▸), using 2 K atoms, 1 Rb atom and a citrate anion as fragments. A Le Bail fit yielded *R*_wp_ = 3.73%. Initial refinement did not include the water mol­ecule, and yielded an acceptable fit (*R*_wp_ = 4.8%), but the *U*_iso_ values of the C atoms in the central part of the mol­ecule were relatively large (∼0.10 Å^2^). The bond-valence sums of the cations were, however, far too low, showing that the water mol­ecule was indeed present. It was inserted in the position from the known monohydrate structures.

The structure was refined by the Rietveld method using *GSAS-II* (Toby & Von Dreele, 2013 ▸). The hydrogen atoms were included in fixed positions, which were recalculated during the course of the refinement using *Materials Studio* (Dassault Systems, 2019 ▸). All C—C and C—O bond distances and all bond angles were restrained based on previous citrate structures: C1—C2 = C4—C5 = 1.51 (1) Å, C2—C3 = C3—C4 = 1.54 (1) Å, C3—C6 = 1.55 (1) Å, C3—O17 = 1.42 (3) Å, C(carbox­yl)—O(carbox­yl) = 1.27 (3) Å, C1—C2—C3 = C3—C4—C5 = 115 (3)°, all angles about C3 = 109 (3)°, carboxyl C—C—O = 115 (3)°, and carboxyl O—C—O = 130 (3)°. Each of the three cation sites was modeled as a mixture of K and Rb; the sums of the site occupancies were constrained to be unity, but the total K and Rb contents were not constrained/restrained, to provide an inter­nal consistency check. The *U*_iso_ of the atoms in the central and outer portions of the citrate anion were constrained to be equal, and the *U*_iso_ of the hydrogen atoms were constrained to be 1.3× those of the atoms to which they are attached. The *U_iso_* of the cations were constrained to be equal. A capillary absorption model (fixed μ.*R* = 0.84, calculated using the tool on the 11-BM web site) was included into the refinement. A Chebyschev polynomial function with four coefficients, along with a peak at 13.11° to model the scattering of the glass capillary, was used to model the background.

Because DFT techniques cannot accommodate disordered systems, three density functional geometry optimizations (with Rb at each of the three cations sites, and K at the other two) were carried out using *CRYSTAL14* (Dovesi *et al.*, 2014 ▸). The basis sets for the H, C, N, and O atoms were those of Gatti *et al.* (1994 ▸), and the basis sets for K and Rb were those of Peintinger *et al.* (2013 ▸). The calculations were run on eight 2.1 GHz Xeon cores (each with 6 Gb RAM) of a 304-core Dell Linux cluster at IIT, using 8 *k*-points and the B3LYP functional.

**Figure 8:**
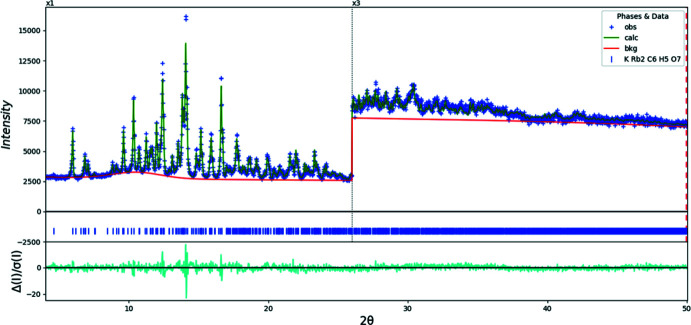
Rietveld plot for (II). The blue crosses represent the observed data points, and the green line is the calculated pattern. The cyan curve is the normalized error plot. The vertical scale has been multiplied by a factor of 3× for 2θ > 26.0°. The row of blue tick marks indicates the calculated reflection positions. The red line is the background curve.

Crystal data, data collection and structure refinement details for (II) are summarized in Table 7 ▸ (Fig. 8 ▸). The same solution and refinement strategy as for (I) was followed. Three density functional geometry optimizations (with K at each of the three cations sites, and Rb at the other two) were carried out using *CRYSTAL17* (Dovesi *et al.*, 2018 ▸) with atom basis sets and computer hardware as described in the previous paragraph.

## Supplementary Material

Crystal structure: contains datablock(s) I, I_19_DFT, I_20_DFT, I_21_DFT, II, II_19_DFT, II_20_DFT, II_21_DFT. DOI: 10.1107/S2056989020011846/hb7917sup1.cif

Supporting information file. DOI: 10.1107/S2056989020011846/hb7917Isup2.cml

Supporting information file. DOI: 10.1107/S2056989020011846/hb7917IIsup3.cml

CCDC references: 2025972, 2025973, 2025974, 2025975, 2025976, 2025977, 2025978, 2025979

Additional supporting information:  crystallographic information; 3D view; checkCIF report

## Figures and Tables

**Table 1:** Hydrogen-bond geometry (Å, °) for (I) *M*19

*D*—H⋯*A*	*D*—H	H⋯*A*	*D*⋯*A*	*D*—H⋯*A*
O17—H18⋯O16	0.98	1.99	2.598	118
O17—H18⋯O13^i^	0.98	2.35	3.196	145
O25—H26⋯O13^ii^	0.99	1.66	2.641	174
O25—H27⋯O15	0.99	1.68	2.662	176

Symmetry codes: (i) 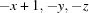; (ii) 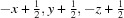.

**Table 2:** Hydrogen-bond geometry (Å, °) for (I) *M*20

*D*—H⋯*A*	*D*—H	H⋯*A*	*D*⋯*A*	*D*—H⋯*A*
O17—H18⋯O16	0.98	1.96	2.598	118
O17—H18⋯O13^i^	0.98	2.37	3.203	142
O25—H26⋯O13^ii^	0.99	1.68	2.662	171
O25—H27⋯O15	0.98	1.72	2.696	176

Symmetry codes: (i) 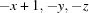; (ii) 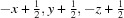.

**Table 3:** Hydrogen-bond geometry (Å, °) for (I) *M*21

*D*—H⋯*A*	*D*—H	H⋯*A*	*D*⋯*A*	*D*—H⋯*A*
O17—H18⋯O16	0.98	1.97	2.594	120
O17—H18⋯O13^i^	0.98	2.33	3.124	138
O25—H26⋯O13^ii^	0.99	1.66	2.643	175
O25—H27⋯O15	0.98	1.71	2.696	175

Symmetry codes: (i) 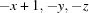; (ii) 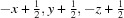.

**Table 4:** Hydrogen-bond geometry (Å, °) for (II) *M*19

*D*—H⋯*A*	*D*—H	H⋯*A*	*D*⋯*A*	*D*—H⋯*A*
O17—H18⋯O16	0.98	1.93	2.579	122
O17—H18⋯O13^i^	0.98	2.45	3.220	136
O25—H26⋯O13^ii^	0.99	1.68	2.660	173
O25—H27⋯O15	0.98	1.72	2.700	174

Symmetry codes: (i) 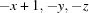; (ii) 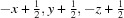.

**Table 5:** Hydrogen-bond geometry (Å, °) for (II) *M*20

*D*—H⋯*A*	*D*—H	H⋯*A*	*D*⋯*A*	*D*—H⋯*A*
O17—H18⋯O16	0.98	1.98	2.596	118
O25—H26⋯O13^i^	0.99	1.66	2.648	177
O25—H27⋯O15	0.99	1.69	2.671	177

Symmetry code: (i) 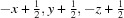.

**Table 6:** Hydrogen-bond geometry (Å, °) for (II) *M*21

*D*—H⋯*A*	*D*—H	H⋯*A*	*D*⋯*A*	*D*—H⋯*A*
O17—H18⋯O16	0.98	1.94	2.582	121
O25—H26⋯O13^i^	0.99	1.66	2.642	173
O25—H27⋯O15	0.98	1.69	2.669	175

Symmetry code: (i) 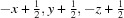.

**Table 7:** Experimental details

	(I)	(II)
Crystal data		
Chemical formula	2K^+^·Rb^+^·C_6_H_5_O_7_^3−^·H_2_O	K^+^·2Rb^+^·C_6_H_5_O_7_^3−^·H_2_O
*M*_r_	365.78	399.97
Crystal system, space group	Monoclinic, *P*2_1_/*n*	Monoclinic, *P*2_1_/*n*
Temperature (K)	300	300
*a*, *b*, *c* (Å)	7.2407 (10), 11.8145 (3), 13.062 (2)	7.3507 (5), 11.8468 (4), 13.2275 (12)
β (°)	98.334 (7)	98.109 (4)
*V* (Å^3^)	1105.56 (9)	1140.37 (6)
*Z*	4	4
Radiation type	*K*α_1,2_, λ = 0.70932, 0.71361 Å	*K*α_1,2_, λ = 0.70932, 0.71361 Å
Specimen shape, size (mm)	Cylinder, 12 × 0.5	Cylinder, 12 × 0.5

Data collection		
Diffractometer	PANalytical Empyrean	PANalytical Empyrean
Specimen mounting	Glass capillary	Glass capillary
Data collection mode	Transmission	Transmission
Scan method	Step	Step
2θ values (°)	2θ_min_ = 1.021 2θ_max_ = 49.985 2θ_step_ = 0.017	2θ_min_ = 1.021 2θ_max_ = 49.985 2θ_step_ = 0.017

Refinement		
*R* factors and goodness of fit	*R*_p_ = 0.026, *R*_wp_ = 0.033, *R*_exp_ = 0.018, *R*(*F* ^2^) = 0.04795, χ^2^ = 3.549	*R*_p_ = 0.024, *R*_wp_ = 0.032, *R*_exp_ = 0.018, *R*(*F* ^2^) = 0.06949, χ^2^ = 3.426
No. of parameters	70	70
No. of restraints	29	29
H-atom treatment	Only H-atom displacement parameters refined	Only H-atom displacement parameters refined
(Δ/σ)_max_	0.205	0.722

Computer programs: *FOX* (Favre-Nicolin & Černý, 2002 ▸), *GSAS*-II (Toby & Von Dreele, 2013 ▸), *Mercury* (Macrae *et al.*, 2020 ▸), *Materials Studio* (Dassault Systems, 2019 ▸), *DIAMOND* (Crystal Impact, 2015 ▸) and *publCIF* (Westrip, 2010 ▸).

## supplementary crystallographic information

### Dipotassium rubidium citrate monohydrate (I) Crystal data

**Table:**

2K^+^·Rb^+^·C_6_H_5_O_7_^3^^−^·H_2_O	*V* = 1105.56 (9) Å^3^
*M**_r_* = 365.78	*Z* = 4
Monoclinic, *P*2_1_/*n*	*D*_x_ = 2.198 Mg m^−^^3^
Hall symbol: -P 2yn	*K*α_1,2_ radiation, λ = 0.70932, 0.71361 Å
*a* = 7.2407 (10) Å	*T* = 300 K
*b* = 11.8145 (3) Å	white
*c* = 13.062 (2) Å	cylinder, 12 × 0.5 mm
β = 98.334 (7)°	Specimen preparation: Prepared at 403 K

### Dipotassium rubidium citrate monohydrate (I) Data collection

**Table:**

PANalytical Empyrean diffractometer	Data collection mode: transmission
Radiation source: sealed X-ray tube	Scan method: step
Specimen mounting: Glass capillary	2θ_min_ = 1.021°, 2θ_max_ = 49.985°, 2θ_step_ = 0.017°

### Dipotassium rubidium citrate monohydrate (I) Refinement

**Table:**

Least-squares matrix: full	29 restraints
*R*_p_ = 0.026	15 constraints
*R*_wp_ = 0.033	Only H-atom displacement parameters refined
*R*_exp_ = 0.018	Weighting scheme based on measured s.u.'s
*R*(*F*^2^) = 0.04795	(Δ/σ)_max_ = 0.205
2931 data points	Background function: Background function: "chebyschev-1" function with 4 terms: 2307(6), -280(6), 22(5), -43(4), Background peak parameters: pos, int, sig, gam: 10.29(6), 1.13(5)e5, 2.80(23)e4, 0.100,
Profile function: Finger-Cox-Jephcoat function parameters U, V, W, X, Y, SH/L: peak variance(Gauss) = Utan(Th)^2^+Vtan(Th)+W: peak HW(Lorentz) = X/cos(Th)+Ytan(Th); SH/L = S/L+H/L U, V, W in (centideg)^2^, X & Y in centideg 19.949, 12.795, 0.000, 2.075, 0.000, 0.032, Crystallite size in microns with "isotropic" model: parameters: Size, G/L mix 1.000, 1.000, Microstrain, "generalized" model (10^6^ * delta Q/Q) parameters: S400, S040, S004, S220, S202, S022, S301, S103, S121, G/L mix 21847.124, 158.305, 5787.320, 10788.591, 22832.702, -379.772, 23098.342, 3225.801, 2172.138, 1.000,	Preferred orientation correction: Spherical Harmonics correction. Order = 2 Model: cylindrical Orientation angles: omega = 0.00; chi = 0.00; phi = 0.00; Coefficients: 0::C(2,0,-2) = -0.0803; 0::C(2,0,0) = 0.1239; 0::C(2,0,2) = 0.2546; Simple spherical harmonic correction Order = 2 Coefficients: 0:0:C(2,-2) = 0.0000; 0:0:C(2,0) = 0.0000; 0:0:C(2,2) = 0.0000
70 parameters	

### Dipotassium rubidium citrate monohydrate (I) Fractional atomic coordinates and isotropic or equivalent isotropic displacement parameters (Å^2^)

**Table:**

	*x*	*y*	*z*	*U*_iso_*/*U*_eq_	Occ. (<1)
C1	0.385 (2)	0.3957 (7)	0.1274 (12)	0.033 (4)*	
C2	0.3114 (18)	0.2874 (6)	0.1675 (13)	0.016 (6)*	
C3	0.4195 (10)	0.1791 (7)	0.1480 (7)	0.016*	
C4	0.3077 (19)	0.0749 (7)	0.1745 (12)	0.016*	
C5	0.358 (2)	−0.0357 (7)	0.1277 (11)	0.033*	
C6	0.6126 (11)	0.1800 (17)	0.2170 (9)	0.033*	
H7	0.31533	0.29370	0.25488	0.021*	
H8	0.15997	0.27640	0.13043	0.021*	
H9	0.32956	0.06167	0.26187	0.021*	
H10	0.15318	0.09193	0.14804	0.021*	
O11	0.267 (3)	0.4511 (11)	0.0657 (13)	0.033*	
O12	0.535 (2)	0.4299 (12)	0.1794 (12)	0.033*	
O13	0.236 (3)	−0.0732 (15)	0.0569 (13)	0.033*	
O14	0.489 (2)	−0.0890 (10)	0.1815 (12)	0.033*	
O15	0.6078 (18)	0.186 (2)	0.3140 (9)	0.033*	
O16	0.7512 (13)	0.1662 (17)	0.1688 (11)	0.033*	
O17	0.4449 (16)	0.1744 (11)	0.0424 (8)	0.033*	
H18	0.51930	0.13650	0.01110	0.043*	
K19	−0.1246 (11)	0.4396 (6)	0.1156 (8)	0.0280 (14)*	0.928
Rb20	−0.1246	0.4396	0.1156	0.0280*	0.072
K21	0.9916 (7)	0.1613 (4)	0.3832 (5)	0.0280*	0.645
Rb22	0.9916	0.1613	0.3832	0.0280*	0.355
K23	0.8338 (8)	−0.0689 (4)	0.1125 (4)	0.0280*	0.534
Rb24	0.8338	−0.0689	0.1125	0.0280*	0.466
O25	0.374 (3)	0.2108 (17)	0.4690 (15)	0.080 (11)*	
H26	0.34120	0.28290	0.45890	0.104*	
H27	0.44720	0.20590	0.41980	0.104*	

### Dipotassium rubidium citrate monohydrate (I) Geometric parameters (Å, º)

**Table:**

C1—C2	1.5094 (19)	K19—O11	3.00 (2)
C1—O11	1.269 (5)	K19—O11^i^	2.763 (16)
C1—O12	1.263 (6)	K19—O12^iv^	2.714 (19)
C2—C1	1.5094 (19)	K19—O14^ix^	2.711 (18)
C2—C3	1.5398 (19)	K19—O15^ix^	3.05 (3)
C3—C2	1.5398 (19)	Rb20—O11	3.00 (2)
C3—C4	1.5398 (19)	Rb20—O11^i^	2.763 (13)
C3—C6	1.5501 (19)	Rb20—O12^iv^	2.714 (17)
C3—O17	1.419 (5)	Rb20—O14^ix^	2.711 (15)
C4—C3	1.5398 (19)	Rb20—O15^ix^	3.05 (2)
C4—C5	1.5100 (19)	Rb20—O25^viii^	2.61 (2)
C5—C4	1.5100 (19)	K21—O11^vii^	3.055 (16)
C5—O13	1.263 (5)	K21—O12^vii^	2.852 (15)
C5—O14	1.261 (5)	K21—O14^ii^	3.077 (14)
C6—C3	1.5501 (19)	K21—O15	2.809 (14)
C6—O15	1.273 (5)	K21—O16	3.076 (16)
C6—O16	1.271 (5)	K21—O17^x^	2.899 (12)
O11—C1	1.269 (5)	K21—O25^iii^	2.89 (2)
O11—K19	3.00 (2)	Rb22—O11^vii^	3.055 (15)
O11—K19^i^	2.763 (16)	Rb22—O12^vii^	2.852 (14)
O11—K21^ii^	3.055 (16)	Rb22—O14^ii^	3.077 (13)
O12—C1	1.263 (6)	Rb22—O15	2.809 (13)
O12—K19^iii^	2.714 (19)	Rb22—O16	3.076 (13)
O12—K21^ii^	2.852 (15)	Rb22—O17^x^	2.899 (11)
O12—K23^ii^	2.746 (15)	Rb22—O25^iii^	2.89 (2)
O13—C5	1.263 (5)	K23—O12^vii^	2.746 (15)
O13—K23^iv^	3.11 (2)	K23—O13^iii^	3.11 (2)
O13—K23^v^	2.766 (19)	K23—O13^v^	2.766 (19)
O14—C5	1.261 (5)	K23—O14	2.784 (16)
O14—K19^vi^	2.711 (18)	K23—O15^vii^	3.07 (2)
O14—K21^vii^	3.077 (14)	K23—O16	2.96 (2)
O14—K23	2.784 (16)	K23—O17^v^	2.922 (14)
O15—C6	1.273 (5)	Rb24—O12^vii^	2.746 (15)
O15—K19^vi^	3.05 (3)	Rb24—O13^iii^	3.11 (2)
O15—K21	2.809 (14)	Rb24—O13^v^	2.766 (19)
O15—K23^ii^	3.07 (2)	Rb24—O14	2.784 (16)
O16—C6	1.271 (5)	Rb24—O15^vii^	3.07 (2)
O16—K21	3.076 (16)	Rb24—O16	2.957 (18)
O16—K23	2.96 (2)	Rb24—O17^v^	2.922 (13)
O17—C3	1.419 (5)	O25—K19^x^	2.61 (2)
O17—K21^viii^	2.899 (12)	O25—K21^iv^	2.89 (2)
O17—K23^v^	2.922 (14)		
			
C2—C1—O11	114.7 (5)	O11^i^—K19—O25^viii^	74.2 (5)
C2—C1—O12	113.9 (5)	O12^iv^—K19—O25^viii^	106.8 (6)
O11—C1—O12	128.8 (5)	O14^ix^—K19—O25^viii^	126.5 (6)
C1—C2—C3	115.7 (5)	K19^i^—K19—O25^viii^	69.1 (5)
C2—C3—C4	109.32 (17)	K21^ix^—K19—O25^viii^	123.5 (5)
C2—C3—C6	109.59 (17)	K21^xi^—K19—O25^viii^	46.3 (4)
C4—C3—C6	109.52 (17)	O11^i^—Rb20—O12^iv^	92.3 (4)
C2—C3—O17	109.46 (17)	O11^i^—Rb20—O14^ix^	159.0 (4)
C4—C3—O17	109.51 (17)	O12^iv^—Rb20—O14^ix^	84.9 (5)
C6—C3—O17	109.43 (17)	O11^i^—Rb20—O25^viii^	74.2 (5)
C3—C4—C5	115.7 (5)	O12^iv^—Rb20—O25^viii^	106.8 (6)
C4—C5—O13	114.5 (5)	O14^ix^—Rb20—O25^viii^	126.5 (5)
C4—C5—O14	114.4 (5)	O12^vii^—Rb22—O15	89.0 (6)
O13—C5—O14	128.3 (5)	O12^vii^—Rb22—O17^x^	146.8 (4)
C3—C6—O15	115.3 (4)	O15—Rb22—O17^x^	87.1 (4)
C3—C6—O16	114.9 (4)	O12^vii^—Rb22—O25^iii^	109.1 (5)
O15—C6—O16	129.5 (5)	O15—Rb22—O25^iii^	162.0 (6)
C1—O11—K19^i^	156.8 (15)	O17^x^—Rb22—O25^iii^	78.0 (4)
C1—O12—K19^iii^	126.7 (14)	O12^vii^—K23—O13^v^	142.4 (5)
C1—O12—K23^ii^	132.9 (11)	O12^vii^—Rb24—O13^v^	142.4 (4)
K19^iii^—O12—K23^ii^	96.1 (6)	O12^vii^—Rb24—O14	82.9 (5)
C5—O13—K23^v^	113.6 (12)	O13^v^—Rb24—O14	104.0 (4)
C5—O14—K19^vi^	128.2 (8)	O12^vii^—Rb24—O17^v^	142.0 (4)
O11^i^—K19—O12^iv^	92.3 (5)	O13^v^—Rb24—O17^v^	71.1 (4)
O11^i^—K19—O14^ix^	159.0 (6)	O14—Rb24—O17^v^	68.0 (4)
O12^iv^—K19—O14^ix^	84.9 (6)		

Symmetry codes: (i) −*x*, −*y*+1, −*z*; (ii) −*x*+3/2, *y*+1/2, −*z*+1/2; (iii) *x*+1, *y*, *z*; (iv) *x*−1, *y*, *z*; (v) −*x*+1, −*y*, −*z*; (vi) −*x*+1/2, *y*−1/2, −*z*+1/2; (vii) −*x*+3/2, *y*−1/2, −*z*+1/2; (viii) *x*+1/2, −*y*+3/2, *z*+1/2; (ix) −*x*+1/2, *y*+1/2, −*z*+1/2; (x) *x*+3/2, −*y*+3/2, *z*+3/2; (xi) *x*−1/2, −*y*+3/2, *z*+1/2.

### (I_19_DFT) Crystal data

**Table:**

C_6_H_7_K_2_O_8_Rb	*b* = 11.8185 Å
*M**_r_* = 370.73	*c* = 13.0617 Å
Monoclinic, *P*2_1_/*n*	β = 98.3340°
Hall symbol: -P 2yn	*V* = 1105.95 Å^3^
*a* = 7.24070 Å	*Z* = 4

### (I_19_DFT) Data collection

**Table:**

*h* = →	*l* = →
*k* = →	

### (I_19_DFT) Fractional atomic coordinates and isotropic or equivalent isotropic displacement parameters (Å^2^)

**Table:**

	*x*	*y*	*z*	*U*_iso_*/*U*_eq_	
C1	0.387602	0.396247	0.134453	0.03300*	
C2	0.300752	0.285078	0.161529	0.01600*	
C3	0.414116	0.177979	0.149608	0.01600*	
C4	0.291736	0.078893	0.178301	0.01600*	
C5	0.334003	−0.034533	0.132030	0.03300*	
C6	0.607979	0.177590	0.217853	0.03300*	
H7	0.270788	0.289262	0.240834	0.02100*	
H8	0.169692	0.273835	0.110202	0.02100*	
H9	0.306865	0.072548	0.262250	0.02100*	
H10	0.146495	0.099677	0.150816	0.02100*	
O11	0.304860	0.450602	0.058045	0.03300*	
O12	0.533995	0.429648	0.191070	0.03300*	
O13	0.215316	−0.069216	0.056951	0.03300*	
O14	0.478172	−0.088049	0.169573	0.03300*	
O15	0.612096	0.186020	0.315093	0.03300*	
O16	0.748786	0.165708	0.173006	0.03300*	
O17	0.441499	0.169428	0.042803	0.03300*	
H18	0.572418	0.148527	0.042548	0.04300*	
Rb19	−0.115564	0.432699	0.114377	0.02800*	
K20	−0.016156	0.159711	0.384860	0.02800*	
K21	0.829410	−0.070765	0.109841	0.02800*	
O25	0.354682	0.211581	0.439575	0.08000*	
H26	0.328247	0.293138	0.446004	0.10400*	
H27	0.445803	0.203599	0.390877	0.10400*	

### (I_19_DFT) Hydrogen-bond geometry (Å, º)

**Table:**

*D*—H···*A*	*D*—H	H···*A*	*D*···*A*	*D*—H···*A*
O17—H18···O16	0.98	1.99	2.598	118
O17—H18···O13^i^	0.98	2.35	3.196	145
O25—H26···O13^ii^	0.99	1.66	2.641	174
O25—H27···O15	0.99	1.68	2.662	176

Symmetry codes: (i) −*x*+1, −*y*, −*z*; (ii) −*x*+1/2, *y*+1/2, −*z*+1/2.

### (I_20_DFT) Crystal data

**Table:**

C_6_H_7_K_2_O_8_Rb	*b* = 11.81851 Å
*M**_r_* = 370.73	*c* = 13.06177 Å
Monoclinic, *P*2_1_/*n*	β = 98.3340°
Hall symbol: -P 2yn	*V* = 1105.95 Å^3^
*a* = 7.24070 Å	*Z* = 4

### (I_20_DFT) Data collection

**Table:**

*h* = →	*l* = →
*k* = →	

### (I_20_DFT) Fractional atomic coordinates and isotropic or equivalent isotropic displacement parameters (Å^2^)

**Table:**

	*x*	*y*	*z*	*U*_iso_*/*U*_eq_	
C1	0.381013	0.394055	0.130708	0.03300*	
C2	0.293184	0.285132	0.162434	0.01600*	
C3	0.402898	0.176581	0.151416	0.01600*	
C4	0.280001	0.078045	0.180294	0.01600*	
C5	0.331506	−0.034836	0.136729	0.03300*	
C6	0.595245	0.173140	0.220658	0.03300*	
H7	0.271064	0.293099	0.242888	0.02100*	
H8	0.157764	0.275194	0.114842	0.02100*	
H9	0.292673	0.072933	0.264274	0.02100*	
H10	0.135438	0.097397	0.149747	0.02100*	
O11	0.281987	0.455743	0.064950	0.03300*	
O12	0.544521	0.418712	0.173263	0.03300*	
O13	0.223087	−0.071101	0.058155	0.03300*	
O14	0.475237	−0.085389	0.180008	0.03300*	
O15	0.598209	0.186110	0.317414	0.03300*	
O16	0.735216	0.152795	0.177058	0.03300*	
O17	0.432382	0.165351	0.045123	0.03300*	
H18	0.563594	0.143536	0.047223	0.04300*	
K19	−0.115017	0.445349	0.113268	0.02800*	
Rb20	−0.002640	0.165932	0.384727	0.02800*	
K21	0.828073	−0.069128	0.116190	0.02800*	
O25	0.395879	0.216870	0.472957	0.08000*	
H26	0.348848	0.295195	0.468149	0.10400*	
H27	0.464202	0.205158	0.414130	0.10400*	

### (I_20_DFT) Hydrogen-bond geometry (Å, º)

**Table:**

*D*—H···*A*	*D*—H	H···*A*	*D*···*A*	*D*—H···*A*
O17—H18···O16	0.98	1.96	2.598	118
O17—H18···O13^i^	0.98	2.37	3.203	142
O25—H26···O13^ii^	0.99	1.68	2.662	171
O25—H27···O15	0.98	1.72	2.696	176

Symmetry codes: (i) −*x*+1, −*y*, −*z*; (ii) −*x*+1/2, *y*+1/2, −*z*+1/2.

### (I_21_DFT) Crystal data

**Table:**

C_6_H_7_K_2_O_8_Rb	*b* = 11.81851 Å
*M**_r_* = 370.73	*c* = 13.06177 Å
Monoclinic, *P*2_1_/*n*	β = 98.3340°
Hall symbol: -P 2yn	*V* = 1105.95 Å^3^
*a* = 7.24070 Å	*Z* = 4

### (I_21_DFT) Data collection

**Table:**

*h* = →	*l* = →
*k* = →	

### (I_21_DFT) Fractional atomic coordinates and isotropic or equivalent isotropic displacement parameters (Å^2^)

**Table:**

	*x*	*y*	*z*	*U*_iso_*/*U*_eq_	
C1	0.382779	0.395000	0.126384	0.03300*	
C2	0.303121	0.285782	0.163456	0.01600*	
C3	0.416131	0.178071	0.152587	0.01600*	
C4	0.292970	0.079198	0.180960	0.01600*	
C5	0.340745	−0.036619	0.141034	0.03300*	
C6	0.608466	0.175258	0.222408	0.03300*	
H7	0.283206	0.295370	0.244113	0.02100*	
H8	0.166028	0.272600	0.118520	0.02100*	
H9	0.302026	0.076170	0.264923	0.02100*	
H10	0.149042	0.098595	0.149226	0.02100*	
O11	0.276815	0.453687	0.061403	0.03300*	
O12	0.547206	0.422937	0.164156	0.03300*	
O13	0.230533	−0.074038	0.063533	0.03300*	
O14	0.479179	−0.089554	0.186680	0.03300*	
O15	0.609842	0.181247	0.319718	0.03300*	
O16	0.750451	0.162898	0.178807	0.03300*	
O17	0.446845	0.169433	0.046354	0.03300*	
H18	0.579348	0.151603	0.047775	0.04300*	
K19	−0.112854	0.436953	0.108689	0.02800*	
K20	−0.012954	0.157061	0.389284	0.02800*	
Rb21	0.837466	−0.072521	0.114962	0.02800*	
O25	0.361121	0.209555	0.452481	0.08000*	
H26	0.324995	0.290235	0.450230	0.10400*	
H27	0.446472	0.197835	0.401325	0.10400*	

### (I_21_DFT) Hydrogen-bond geometry (Å, º)

**Table:**

*D*—H···*A*	*D*—H	H···*A*	*D*···*A*	*D*—H···*A*
O17—H18···O16	0.98	1.97	2.594	120
O17—H18···O13^i^	0.98	2.33	3.124	138
O25—H26···O13^ii^	0.99	1.66	2.643	175
O25—H27···O15	0.98	1.71	2.696	175

Symmetry codes: (i) −*x*+1, −*y*, −*z*; (ii) −*x*+1/2, *y*+1/2, −*z*+1/2.

### Potassium dirubidium citrate monohydrate (II) Crystal data

**Table:**

K^+^·2Rb^+^·C_6_H_5_O_7_^3^^−^·H_2_O	*V* = 1140.37 (6) Å^3^
*M**_r_* = 399.97	*Z* = 4
Monoclinic, *P*2_1_/*n*	*D*_x_ = 2.330 Mg m^−^^3^
Hall symbol: -P 2yn	*K*α_1,2_ radiation, λ = 0.70932, 0.71361 Å
*a* = 7.3507 (5) Å	*T* = 300 K
*b* = 11.8468 (4) Å	white
*c* = 13.2275 (12) Å	cylinder, 12 × 0.5 mm
β = 98.109 (4)°	Specimen preparation: Prepared at 403 K

### Potassium dirubidium citrate monohydrate (II) Data collection

**Table:**

PANalytical Empyrean diffractometer	Data collection mode: transmission
Radiation source: sealed X-ray tube	Scan method: step
Specimen mounting: glass capillary	2θ_min_ = 1.021°, 2θ_max_ = 49.985°, 2θ_step_ = 0.017°

### Potassium dirubidium citrate monohydrate (II) Refinement

**Table:**

Least-squares matrix: full	29 restraints
*R*_p_ = 0.024	15 constraints
*R*_wp_ = 0.032	Only H-atom displacement parameters refined
*R*_exp_ = 0.018	Weighting scheme based on measured s.u.'s
*R*(*F*^2^) = 0.06949	(Δ/σ)_max_ = 0.722
2931 data points	Background function: Background function: "chebyschev-1" function with 4 terms: 2587(5), -233(6), 10(4), -28(4), Background peak parameters: pos, int, sig, gam: 10.54(5), 1.39(5)e5, 3.08(20)e4, 0.100,
Profile function: Finger-Cox-Jephcoat function parameters U, V, W, X, Y, SH/L: peak variance(Gauss) = Utan(Th)^2^+Vtan(Th)+W: peak HW(Lorentz) = X/cos(Th)+Ytan(Th); SH/L = S/L+H/L U, V, W in (centideg)^2^, X & Y in centideg 19.949, 12.795, 0.000, 2.075, 0.000, 0.032, Crystallite size in microns with "isotropic" model: parameters: Size, G/L mix 1.000, 1.000, Microstrain, "generalized" model (10^6^ * delta Q/Q) parameters: S400, S040, S004, S220, S202, S022, S301, S103, S121, G/L mix 629.894, 666.269, 773.433, -118.805, -89.875, -165.278, 167.735, 57.322, 73.783, 1.000,	Preferred orientation correction: Spherical Harmonics correction. Order = 2 Model: cylindrical Orientation angles: omega = 0.00; chi = 0.00; phi = 0.00; Coefficients: 0::C(2,0,-2) = -0.0045; 0::C(2,0,0) = 0.1825; 0::C(2,0,2) = 0.1091; March-Dollase correction coef. = 1.000 axis = [0, 0, 1]
70 parameters	

### Potassium dirubidium citrate monohydrate (II) Fractional atomic coordinates and isotropic or equivalent isotropic displacement parameters (Å^2^)

**Table:**

	*x*	*y*	*z*	*U*_iso_*/*U*_eq_	Occ. (<1)
C1	0.395 (3)	0.3900 (9)	0.1268 (15)	0.035 (5)*	
C2	0.317 (2)	0.2852 (8)	0.1695 (15)	0.022 (8)*	
C3	0.4170 (10)	0.1747 (9)	0.1508 (8)	0.022*	
C4	0.303 (2)	0.0734 (8)	0.1788 (14)	0.022*	
C5	0.348 (2)	−0.0379 (9)	0.1324 (16)	0.035*	
C6	0.6080 (12)	0.173 (2)	0.2178 (11)	0.035*	
H7	0.32318	0.29412	0.25575	0.028*	
H8	0.16642	0.27663	0.13442	0.028*	
H9	0.32518	0.06108	0.26513	0.028*	
H10	0.15106	0.09267	0.15353	0.028*	
O11	0.280 (3)	0.4464 (15)	0.0661 (16)	0.035*	
O12	0.552 (3)	0.4191 (15)	0.1714 (16)	0.035*	
O13	0.229 (3)	−0.0728 (16)	0.0599 (16)	0.035*	
O14	0.489 (3)	−0.0872 (13)	0.1775 (16)	0.035*	
O15	0.606 (2)	0.188 (3)	0.3132 (11)	0.035*	
O16	0.7441 (14)	0.167 (2)	0.1683 (15)	0.035*	
O17	0.4399 (19)	0.1676 (15)	0.0464 (9)	0.035*	
H18	0.51790	0.14150	0.01080	0.046*	
K19	−0.1195 (9)	0.4371 (6)	0.1136 (6)	0.0293 (13)*	0.621
Rb20	−0.1195	0.4371	0.1136	0.0293*	0.379
K21	0.9951 (6)	0.1642 (5)	0.3815 (5)	0.0293*	0.394
Rb22	0.9951	0.1642	0.3815	0.0293*	0.606
K23	0.8366 (8)	−0.0671 (5)	0.1150 (5)	0.0293*	0.356
Rb24	0.8366	−0.0671	0.1150	0.0293*	0.644
O25	0.387 (4)	0.205 (3)	0.456 (2)	0.122 (15)*	
H26	0.34760	0.27190	0.45840	0.159*	
H27	0.46070	0.20620	0.41170	0.159*	

### Potassium dirubidium citrate monohydrate (II) Geometric parameters (Å, º)

**Table:**

C1—C2	1.5099 (18)	O17—K23^v^	2.981 (16)
C1—O11	1.270 (5)	K19—O11	3.09 (3)
C1—O12	1.267 (5)	K19—O11^i^	2.855 (17)
C2—C1	1.5099 (18)	K19—O12^iv^	2.65 (2)
C2—C3	1.5403 (18)	K19—O14^ix^	2.81 (2)
C3—C2	1.5403 (18)	K19—O25^viii^	2.69 (3)
C3—C4	1.5402 (18)	Rb20—O11	3.09 (2)
C3—C6	1.5508 (18)	Rb20—O11^i^	2.855 (16)
C3—O17	1.417 (5)	Rb20—O12^iv^	2.65 (2)
C4—C3	1.5402 (18)	Rb20—O14^ix^	2.81 (2)
C4—C5	1.5104 (18)	Rb20—O15^ix^	3.12 (3)
C5—C4	1.5104 (18)	Rb20—O25^viii^	2.69 (3)
C5—O13	1.270 (5)	K21—O11^vii^	3.09 (2)
C5—O14	1.264 (5)	K21—O12^vii^	2.995 (19)
C6—C3	1.5508 (18)	K21—O14^ii^	3.053 (17)
C6—O15	1.277 (5)	K21—O15	2.890 (16)
C6—O16	1.272 (5)	K21—O17^x^	3.024 (15)
O11—C1	1.270 (5)	K21—O25^iii^	2.95 (3)
O11—K19	3.09 (3)	Rb22—O11^vii^	3.092 (19)
O11—K19^i^	2.855 (17)	Rb22—O12^vii^	2.995 (19)
O11—K21^ii^	3.09 (2)	Rb22—O14^ii^	3.053 (16)
O12—C1	1.267 (5)	Rb22—O15	2.890 (15)
O12—K19^iii^	2.65 (2)	Rb22—O16	3.145 (17)
O12—K21^ii^	2.995 (19)	Rb22—O17^x^	3.024 (14)
O12—K23^ii^	2.83 (2)	Rb22—O25^iii^	2.95 (3)
O13—C5	1.270 (5)	K23—O12^vii^	2.83 (2)
O13—K23^iv^	3.08 (3)	K23—O13^iii^	3.08 (3)
O13—K23^v^	2.83 (2)	K23—O13^v^	2.83 (2)
O14—C5	1.264 (5)	K23—O14	2.81 (2)
O14—K19^vi^	2.81 (2)	K23—O15^vii^	3.06 (3)
O14—K21^vii^	3.053 (17)	K23—O16	2.96 (2)
O14—K23	2.81 (2)	K23—O17^v^	2.981 (16)
O15—C6	1.277 (5)	Rb24—O12^vii^	2.83 (2)
O15—Rb20^vi^	3.12 (3)	Rb24—O13^iii^	3.08 (3)
O15—K21	2.890 (16)	Rb24—O13^v^	2.83 (2)
O15—K23^ii^	3.06 (3)	Rb24—O14	2.81 (2)
O16—C6	1.272 (5)	Rb24—O15^vii^	3.06 (3)
O16—Rb22	3.145 (17)	Rb24—O16	2.96 (2)
O16—K23	2.96 (2)	Rb24—O17^v^	2.981 (15)
O17—C3	1.417 (5)	O25—K19^x^	2.69 (3)
O17—K21^viii^	3.024 (15)	O25—K21^iv^	2.95 (3)
			
C2—C1—O11	114.5 (5)	O15—C6—O16	129.5 (5)
C2—C1—O12	114.7 (5)	C1—O12—K19^iii^	134.2 (17)
O11—C1—O12	129.2 (5)	O12^iv^—K19—O25^viii^	107.0 (8)
C1—C2—C3	115.05 (18)	O11^i^—Rb20—O12^iv^	89.6 (5)
C2—C3—C4	109.49 (17)	O11^i^—Rb20—O14^ix^	155.8 (6)
C2—C3—C6	109.51 (17)	O12^iv^—Rb20—O14^ix^	84.7 (6)
C4—C3—C6	109.52 (17)	O11^i^—Rb20—O25^viii^	72.9 (7)
C2—C3—O17	109.43 (17)	O12^iv^—Rb20—O25^viii^	107.0 (8)
C4—C3—O17	109.41 (17)	O14^ix^—Rb20—O25^viii^	131.2 (7)
C6—C3—O17	109.47 (17)	K19^i^—Rb20—O25^viii^	65.7 (7)
C3—C4—C5	115.04 (18)	K21^ix^—Rb20—O25^viii^	122.7 (6)
C4—C5—O13	115.1 (5)	K21^xi^—Rb20—O25^viii^	46.7 (6)
C4—C5—O14	115.1 (5)	K23^ix^—Rb20—O25^viii^	136.0 (7)
O13—C5—O14	129.4 (4)	O12^vii^—Rb24—O13^v^	147.2 (5)
C3—C6—O15	115.3 (5)	O12^vii^—Rb24—O14	81.4 (6)
C3—C6—O16	114.9 (5)	O13^v^—Rb24—O14	103.4 (5)

Symmetry codes: (i) −*x*, −*y*+1, −*z*; (ii) −*x*+3/2, *y*+1/2, −*z*+1/2; (iii) *x*+1, *y*, *z*; (iv) *x*−1, *y*, *z*; (v) −*x*+1, −*y*, −*z*; (vi) −*x*+1/2, *y*−1/2, −*z*+1/2; (vii) −*x*+3/2, *y*−1/2, −*z*+1/2; (viii) *x*+1/2, −*y*+3/2, *z*+1/2; (ix) −*x*+1/2, *y*+1/2, −*z*+1/2; (x) *x*+3/2, −*y*+3/2, *z*+3/2; (xi) *x*−1/2, −*y*+3/2, *z*+1/2.

### (II_19_DFT) Crystal data

**Table:**

C_6_H_7_KO_8_Rb_2_	*b* = 11.846841 Å
*M**_r_* = 417.10	*c* = 13.227547 Å
Monoclinic, *P*2_1_/*n*	β = 98.1091°
Hall symbol: -P 2yn	*V* = 1140.37 Å^3^
*a* = 7.350692 Å	*Z* = 4

### (II_19_DFT) Data collection

**Table:**

*h* = →	*l* = →
*k* = →	

### (II_19_DFT) Fractional atomic coordinates and isotropic or equivalent isotropic displacement parameters (Å^2^)

**Table:**

	*x*	*y*	*z*	*U*_iso_*/*U*_eq_	
C1	0.379147	0.393481	0.127092	0.03533*	
C2	0.297157	0.284667	0.161900	0.02188*	
C3	0.408299	0.176847	0.153108	0.02188*	
C4	0.288255	0.078471	0.182726	0.02188*	
C5	0.337989	−0.036751	0.142957	0.03533*	
C6	0.597428	0.175190	0.221702	0.03533*	
H7	0.274230	0.294909	0.241047	0.02844*	
H8	0.163702	0.272328	0.115834	0.02844*	
H9	0.299322	0.075476	0.265689	0.02844*	
H10	0.146079	0.097106	0.152122	0.02844*	
O11	0.275146	0.455071	0.065511	0.03533*	
O12	0.542964	0.417971	0.163384	0.03533*	
O13	0.230948	−0.074562	0.066665	0.03533*	
O14	0.476999	−0.086921	0.187735	0.03533*	
O15	0.599178	0.183712	0.317526	0.03533*	
O16	0.736783	0.160796	0.178156	0.03533*	
O17	0.439851	0.165051	0.048570	0.03533*	
H18	0.571275	0.148706	0.051908	0.04593*	
K19	−0.115873	0.442248	0.109784	0.02929*	
Rb20	0.997078	0.165805	0.384557	0.02929*	
Rb21	0.835191	−0.069923	0.115567	0.02929*	
O25	0.393473	0.216240	0.468716	0.12179*	
H26	0.342581	0.293118	0.460336	0.15833*	
H27	0.461303	0.202302	0.411083	0.58330*	

### (II_19_DFT) Hydrogen-bond geometry (Å, º)

**Table:**

*D*—H···*A*	*D*—H	H···*A*	*D*···*A*	*D*—H···*A*
O17—H18···O16	0.98	1.93	2.579	122
O17—H18···O13^i^	0.98	2.45	3.220	136
O25—H26···O13^ii^	0.99	1.68	2.660	173
O25—H27···O15	0.98	1.72	2.700	174

Symmetry codes: (i) −*x*+1, −*y*, −*z*; (ii) −*x*+1/2, *y*+1/2, −*z*+1/2.

### (II_20_DFT) Crystal data

**Table:**

C_6_H_7_KO_8_Rb_2_	*b* = 11.846841 Å
*M**_r_* = 417.10	*c* = 13.227549 Å
Monoclinic, *P*2_1_/*n*	β = 98.1091°
Hall symbol: -P 2yn	*V* = 1140.37 Å^3^
*a* = 7.350692 Å	*Z* = 4

### (II_20_DFT) Data collection

**Table:**

*h* = →	*l* = →
*k* = →	

### (II_20_DFT) Fractional atomic coordinates and isotropic or equivalent isotropic displacement parameters (Å^2^)

**Table:**

	*x*	*y*	*z*	*U*_iso_*/*U*_eq_	
C1	0.384218	0.392861	0.126973	0.03533*	
C2	0.305212	0.282429	0.160374	0.02188*	
C3	0.419109	0.170061	0.149253	0.02188*	
C4	0.298983	0.076671	0.177466	0.02188*	
C5	0.342101	−0.038417	0.135603	0.03533*	
C6	0.608783	0.176423	0.217478	0.03533*	
H7	0.280007	0.289357	0.239365	0.02844*	
H8	0.173856	0.268336	0.112922	0.02844*	
H9	0.309871	0.072365	0.260330	0.02844*	
H10	0.156529	0.096601	0.148599	0.02844*	
O11	0.294864	0.443191	0.051752	0.03533*	
O12	0.531397	0.429050	0.177561	0.03533*	
O13	0.227200	−0.075640	0.061912	0.03533*	
O14	0.481315	−0.091449	0.176152	0.03533*	
O15	0.610259	0.180902	0.313492	0.03533*	
O16	0.749135	0.169196	0.173663	0.03533*	
O17	0.448775	0.168279	0.044235	0.03533*	
H18	0.579224	0.151337	0.044285	0.04593*	
Rb19	−0.113845	0.425126	0.111367	0.02929*	
K20	0.978236	0.156277	0.385143	0.02929*	
Rb21	0.839459	−0.073164	0.110994	0.02929*	
O25	0.343562	0.206112	0.429428	0.12179*	
H26	0.316358	0.287413	0.435388	0.15833*	
H27	0.438648	0.197642	0.384416	0.5833*	

### (II_20_DFT) Hydrogen-bond geometry (Å, º)

**Table:**

*D*—H···*A*	*D*—H	H···*A*	*D*···*A*	*D*—H···*A*
O17—H18···O16	0.98	1.98	2.596	118
O25—H26···O13^i^	0.99	1.66	2.648	177
O25—H27···O15	0.99	1.69	2.671	177

Symmetry code: (i) −*x*+1/2, *y*+1/2, −*z*+1/2.

### (II_21_DFT) Crystal data

**Table:**

C_6_H_7_KO_8_Rb_2_	*b* = 11.846841 Å
*M**_r_* = 417.10	*c* = 13.227549 Å
Monoclinic, *P*2_1_/*n*	β = 98.1091°
Hall symbol: -P 2yn	*V* = 1140.37 Å^3^
*a* = 7.350692 Å	*Z* = 4

### (II_21_DFT) Data collection

**Table:**

*h* = →	*l* = →
*k* = →	

### (II_21_DFT) Fractional atomic coordinates and isotropic or equivalent isotropic displacement parameters (Å^2^)

**Table:**

	*x*	*y*	*z*	*U*_iso_*/*U*_eq_	
C1	0.384640	0.395404	0.135647	0.03533*	
C2	0.297849	0.285289	0.163395	0.02188*	
C3	0.407572	0.177871	0.151465	0.02188*	
C4	0.288381	0.079456	0.181825	0.02188*	
C5	0.330118	−0.032430	0.134234	0.03533*	
C6	0.599433	0.176950	0.217300	0.03533*	
H7	0.271618	0.290599	0.242332	0.02844*	
H8	0.167350	0.274630	0.114157	0.02844*	
H9	0.307767	0.072515	0.264723	0.02844*	
H10	0.144764	0.100005	0.156692	0.02844*	
O11	0.298137	0.452327	0.063566	0.03533*	
O12	0.534747	0.425208	0.187730	0.03533*	
O13	0.213385	−0.065891	0.060067	0.03533*	
O14	0.473694	−0.085091	0.170046	0.03533*	
O15	0.605990	0.187134	0.313101	0.03533*	
O16	0.736153	0.162119	0.171402	0.03533*	
O17	0.433433	0.166966	0.045915	0.03533*	
H18	0.563251	0.147163	0.047130	0.04593*	
Rb19	−0.115589	0.436888	0.115484	0.02929*	
Rb20	0.997021	0.166511	0.381875	0.02929*	
K21	0.826737	−0.066349	0.111425	0.02929*	
O25	0.389886	0.220556	0.456407	0.12179*	
H26	0.349411	0.300046	0.455368	0.15833*	
H27	0.463621	0.210092	0.400608	0.15833*	

### (II_21_DFT) Hydrogen-bond geometry (Å, º)

**Table:**

*D*—H···*A*	*D*—H	H···*A*	*D*···*A*	*D*—H···*A*
O17—H18···O16	0.98	1.94	2.582	121
O25—H26···O13^i^	0.99	1.66	2.642	173
O25—H27···O15	0.98	1.69	2.669	175

Symmetry code: (i) −*x*+1/2, *y*+1/2, −*z*+1/2.
